# Alanine dehydrogenases from four different microorganisms: characterization and their application in L-alanine production

**DOI:** 10.1186/s13068-023-02373-5

**Published:** 2023-08-03

**Authors:** Pengfei Gu, Qianqian Ma, Shuo Zhao, Qiang Li, Juan Gao

**Affiliations:** School of Biological Science and Technology, University of Jinan, Jinan, 250022 People’s Republic of China

**Keywords:** Alanine dehydrogenase, L-alanine, *Escherichia coli*, Metabolic engineering

## Abstract

**Background:**

Alanine dehydrogenase (AlaDH) belongs to oxidoreductases, and it exists in several different bacteria species and plays a key role in microbial carbon and nitrogen metabolism, spore formation and photosynthesis. In addition, AlaDH can also be applied in biosynthesis of L-alanine from cheap carbon source, such as glucose.

**Results:**

To achieve a better performance of L-alanine accumulation, system evaluation and comparison of different AlaDH with potential application value are essential. In this study, enzymatic properties of AlaDH from *Bacillus subtilis* 168 (BsAlaDH), *Bacillus cereus* (BcAlaDH), *Mycobacterium smegmatis* MC^2^ 155 (MsAlaDH) and *Geobacillus stearothermophilus* (GsAlaDH) were firstly carefully investigated. Four different AlaDHs have few similarities in optimum temperature and optimum pH, while they also exhibited significant differences in enzyme activity, substrate affinity and enzymatic reaction rate. The wild *E. coli* BL21 with these four AlaDHs could produce 7.19 g/L, 7.81 g/L, 6.39 g/L and 6.52 g/L of L-alanine from 20 g/L glucose, respectively. To further increase the L-alanine titer, competitive pathways for L-alanine synthesis were completely blocked in *E. coli*. The final strain M-6 could produce 80.46 g/L of L-alanine with a yield of 1.02 g/g glucose after 63 h fed-batch fermentation, representing the highest yield for microbial L-alanine production.

**Conclusions:**

Enzyme assay, biochemical characterization and structure analysis of BsAlaDH, BcAlaDH, MsAlaDH and GsAlaDH were carried out. In addition, application potential of these four AlaDHs in L-alanine productions were explored. The strategies here can be applied for developing L-alanine producing strains with high titers.

**Supplementary Information:**

The online version contains supplementary material available at 10.1186/s13068-023-02373-5.

## Introduction

Alanine dehydrogenase (AlaDH; EC 1.4.1.1), belongs to amino acid dehydrogenase, can catalyze the oxidation of alanine with NAD^+^ (NADP^+^) as coenzyme and the reduction of pyruvate with NADH (NADPH) as coenzyme [1]. The AlaDH is widely distributed in many organisms, such as *Rhodobacter capsulatus* [2], *Streptomyces fradiae* [3], *Archaeoglobus fulgidus* [4] and *Mycobacterium tuberculosis* [5]. To date, AlaDHs have been found to be involved in several microbial physiological processes. For example, AlaDH is essential for the growth and sporulation of *Bacillus subtilis* [1], and it can also regulate the steady-state balance of ketoacids and amino acids [6, 7]. In addition, AlaDH can acted as antigen in pathogenic bacteria, such as *Mycobacterium tuberculosis* [8].

The common natural substrates of AlaDH are L-alanine or pyruvate. In common, the specific activities of pyruvate reductive aminase of AlaDH were relatively higher than these of alanine oxidative dehydrogenase activity [9, 10]. In addition, a few fungal AlaDH exhibit broad substrate specificity of oxidative deamination, such as L-alanine, L-serine, L-isoleucine, and L-threonine. An archaeal AlaDH from *Archaeoglobus fulgidus* was also discovered to possess selective activities for L-alanine and L-2-aminobutyrate and a lower activity toward several amino acids [11].

L-alanine is one of the smallest chiral compounds, which is widely applied in the field of food, pharmaceutical and veterinary. L-alanine can also be used as a feedstock for the synthesis of thermoplastics, such as polyamides [12]. In addition, L-alanine is one of the most important raw materials for methylglycinediacetic acid, which is a green chelating agent with excellent performance [13]. However, relatively high production cost of traditional method such as enzymic decarboxylation of L-aspartic acid limits its widely application. To overcome obstacle of production cost, development of a renewable and low-cost production strategy is desired.

Accordingly, microbial production of L-alanine from cheap carbon source became an important option. In some microorganisms, such as *Arthrobacter oxydans* [14], *Bacillus sphaericus* [15] and *Clostridium* sp. P2 [16], L-alanine could be directly synthesized by a reduced nicotinamide adenine dinucleotide (NADH)-linked AlaDH. In other hand, *Escherichia coli*, a model microorganism that possesses clear genetic background, diverse engineering tools and fast growth in cheap media, has been widely applied in production of valuable compounds [17, 18]. Unfortunately, no endogenous AlaDH was found in wild *E. coli*. Taken together, introduction of heterogeneous AlaDH into *E. coli* can achieve a robust production of L-alanine from cheap carbon source, and screening of AlaDH with excellent enzyme property appears to be particularly important.

In this study, enzymatic properties of AlaDH from *Bacillus subtilis* 168 (BsAlaDH), *Bacillus cereus* (BcAlaDH), *Mycobacterium smegmatis* MC^2^ 155 (MsAlaDH) and *Geobacillus stearothermophilus* (GsAlaDH) were carefully investigated and compared. In addition, the effect of BsAlaDH, BcAlaDH, MsAlaDH and GsAlaDH for L-alanine production in *E. coli* were also explored, obtaining a maximum of 80.46 g/L g/L L-alanine by fed-batch fermentation.

## Results and discussion

### Multiple sequence alignment and phylogenetic tree analysis

**Fig. 1:**
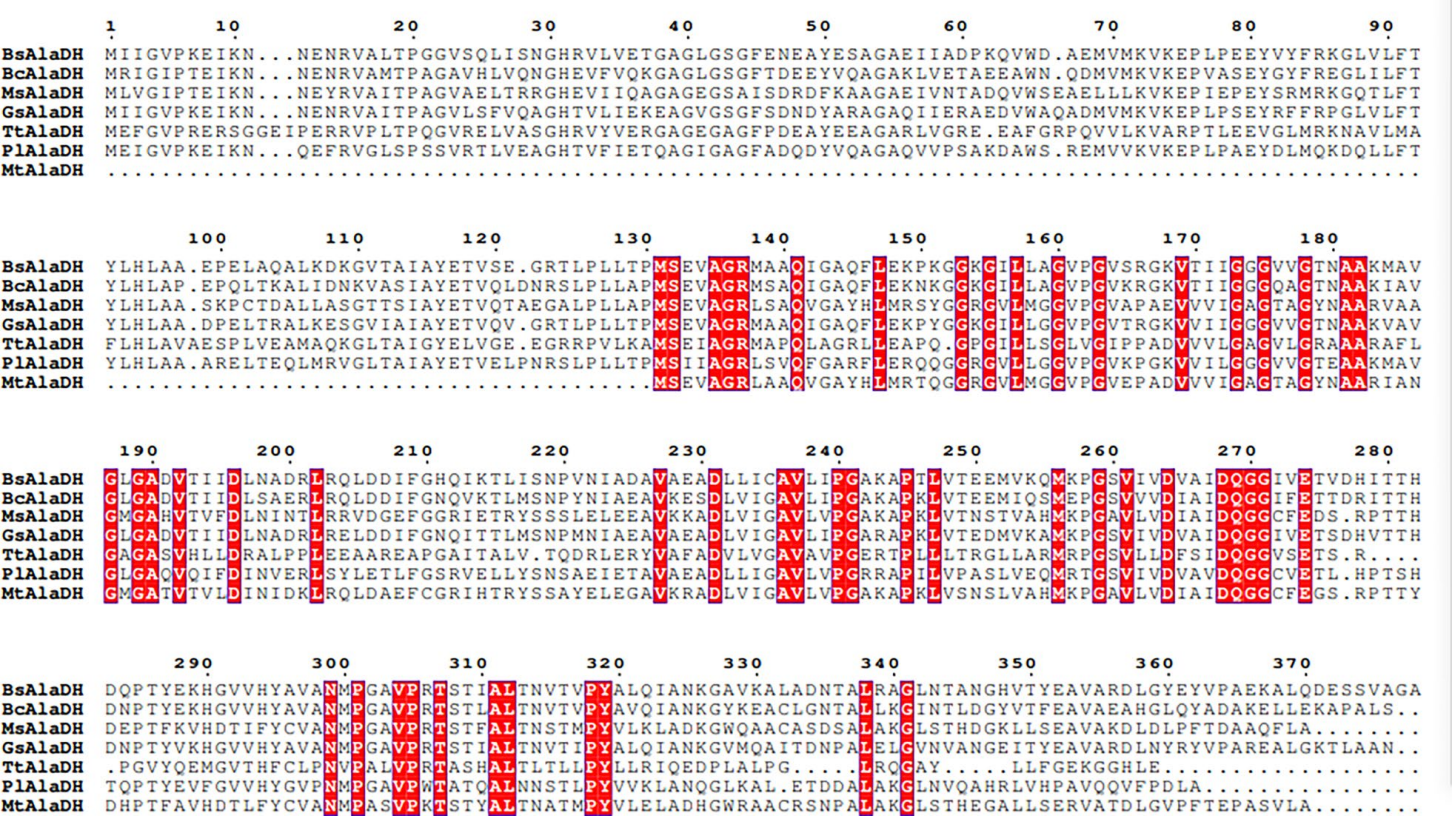
Multiple sequence alignment of AlaDH from *Bacillus subtilis* 168 (BsAlaDH), *Bacillus cereus* (BcAlaDH), *Mycobacterium smegmatis* MC^2^ 155 (MsAlaDH), *Geobacillus stearothermophilus* (GsAlaDH), *Themus thermophilus* (TtAlaDH), *Photoidium lapidum* (PlAlaDH)and *Mycobacterium tuberculosis* H37Rv (MtAlaDH). The sequence alignment was performed using the ClustalX program and exhibited by Expript 3.0

As AlaDHs originating from *B. subtilis* 168, *B. cereus*, *M. smegmatis* MC^2^ 155 and *G. stearothermophilus* have not been carefully analyzed and compared with each other in previous study, these four AlaDHs were selected as candidates in this work. By carefully screening of CAZy database, AlaDH from *Themus thermophilus* (TtAlaDH), *Photoidium lapidum* (PlAlaDH) and *Mycobacterium tuberculosis* H37Rv (MtAlaDH) were selected as templates to analyze protein sequence homology with BsAlaDH, BcAlaDH, MsAlaDH and GsAlaDH. As shown in Fig. 1, a total of 52 invariant residues were found in protein sequences of these seven AlaDHs, and parts of invariant residues were clustered in the crack between two domains of the subunit, which are usually named Domain I and Domain II, respectively. The Domain I and Domain II are connected by an α-helix. The NAD^+^ bounded region is located at the C-terminal end of Domain I, and pyruvate bounded region is generally located in the crack of Domain I and Domain II. Therefore, the active site is most likely located in this region. His95 and Asp270, close to the carbonyl oxygen atom of pyruvate, were considered as active-site residues and participated in proton transfer steps during catalysis for AlaDH from *Mycobacterium tuberculosis* [19]. Otherwise, Asp270 can also move the domain close to nicotinamide ring and pyruvate, resulting a shorter distance between His95 and Asp270. In addition, pyruvate is closely bound to the side chains of two residues Arg15 and Lys75. In a previous report, AlaDH activity was completely lost after replacing four conservative residues Arg15, Lys75, His96 and Asp269 with Ala, indicating the significance of these residues for AlaDH activity [20]. In addition, AlaDH from *M. tuberculosis* (MtAlaDH) was found to transform its conformation from open to closed state when NADH binding to the active site by molecular dynamics simulation, even though mutation occurred in its conservative residue [21, 22]. This phenomenon was possibly due to Asp270 maintained the stability of nicotinamide ring and ribose of NADH through hydrogen bond interaction, while His96 was involved in the structural rearrangement of active sites and loss of catalytic activity. In addition, Met301 was predicted to play a major role in the catalytic reaction of MtAlaDH by fixing the position of NADH nicotinamide ring and preventing the rotation of NADH.

**Fig. 2:**
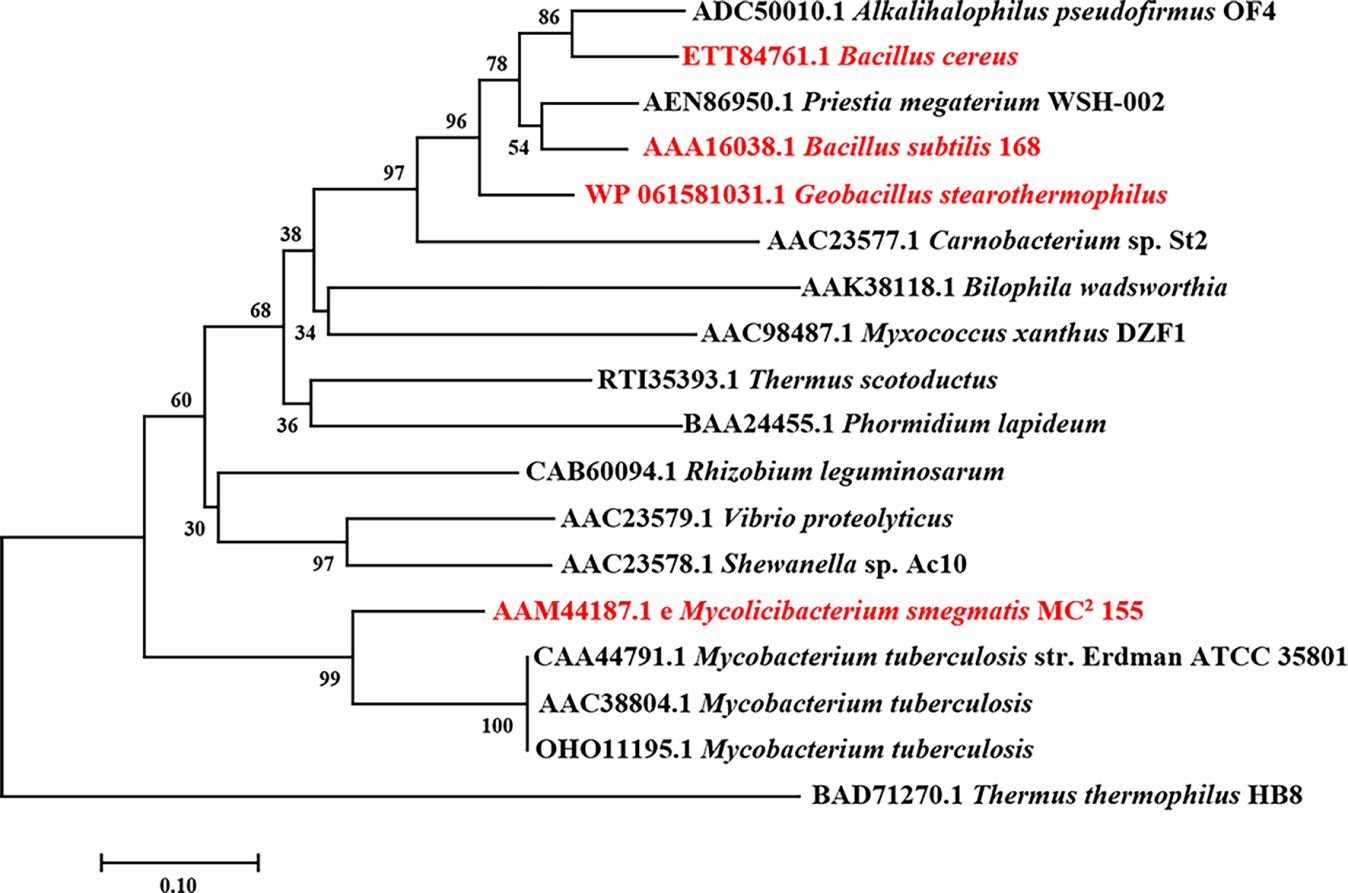
Phylogenetic tree analysis of AlaDHs

And then, phylogenetic tree of AlaDH from numerous different bacteria was analyzed and shown in Fig. 2, and BcAlaDH, BsAlaDH, GsAlaDH and MtAlaDH employed in this study were colored in red. The BcAlaDH, BsAlaDH and GsAlaDH have high similarity with each other, while MsAlaDH exhibited more closely related to MtAlaDH than the other three AlaDHs. This result is broadly consistent with the phylogenetic relationship of *B.subtilis*, *B. cereus*, *M. smegmatis* and *G. stearothermophilus*.

### Expression and purification of AlaDH

**Fig. 3:**
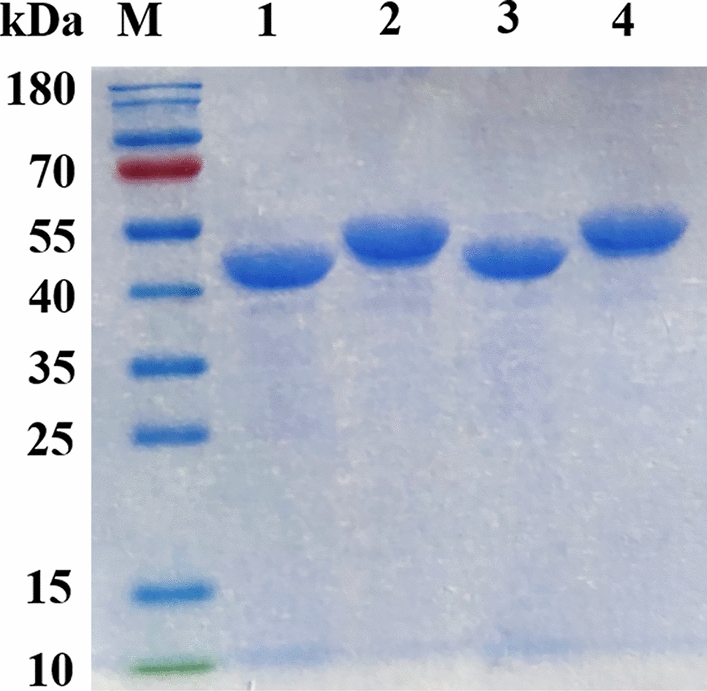
SDS-PAGE of AlaDHs. (M Protein marker; 1 BsAlaDH; 2 BcAlaDH; 3 GsAlaDH; 4 MsAlaDH)

Four *ald* genes encoding AlaDH derived from *B. subtilis* 168, *B. cereus*, *M. smegmatis* MC^2^ 155 and *G. stearothermophilus* were ligated into pET28a and then transformed into *E. coli* BL21 (DE3). As shown in Fig. 3, all of four AlaDHs purified by Ni^2+^ affinity chromatography exhibited obvious bands at about 45–55 kDa by SDS-PAGE gel electrophoresis. Among them, the protein size of BcAlaDH was a little larger than the other AlaDH. This slight distinction in protein size was also reported in other AlaDHs, such as AlaDH from *Shewanella* sp.AC10 (43 kDa) [23], *Thermus thermophilus* (48 kDa) [24], *Mycobacterium tuberculosis* (40 kDa) [25], *Pseudomonas* sp (about 53 kDa) [26] and *Streptomyces fradiae* (about 51 kDa) [3].

### Structure analysis and evaluation of AlaDH

The AlaDH from *M. tuberculosis* H37Rv (PDB ID: 2VOE) possessing relatively high sequence homology with BsAlaDH (51.09%), BcAlaDH (54.05%), MsAlaDH (80.59%) and GsAlaDH (52.97%) was selected as a template. Structural models of four AlaDH were built by SWISS-MODEL, and the reliability was evaluated according to the Laplace conformation map. As shown in Additional file 1: Fig. S1, 92.78%, 92.91%, 94.08% and 92.78% of the residues are located in the most favorable region for BsAlaDH, BcAlaDH, MsAlaDH and GsAlaDH separately, while 5.61%, 4.70%, 8.66% and 5.61% of the residues are located in the allowable region. In contrast, very few residues are located in the non-allowable region, indicating that the current model is reasonable.

**Fig. 4:**
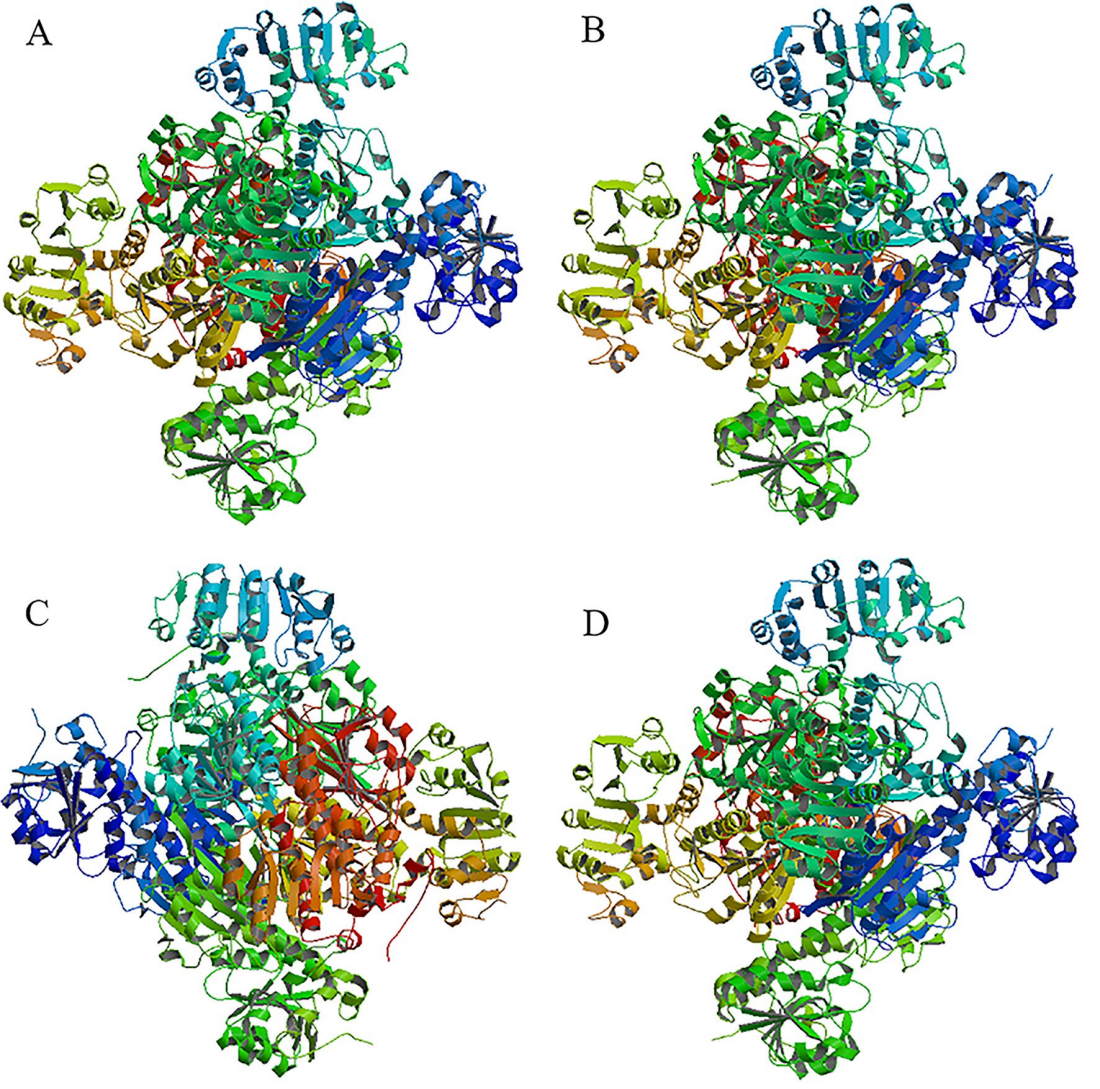
SWISS-MODEL predicts the three-dimensional structures of (**A**) BsAlaDH (**B**) BcAlaDH (**C**) MsAlaDH and (**D**) GsAlaDH. The AlaDH from *M. tuberculosis* H37Rv (PDB ID: 2VOE) was selected as a template

The three-dimensional structures of BsAlaDH, BcAlaDH, MsAlaDH and GsAlaDH suggested all of them were possible hexamers (Fig. 4), which were similar with previous reports [27]. AlaDH mainly contains a NAD binding domain composed of a large number of C-terminal residues and a catalytic domain for pyruvate binding. The two domains are separated by an α-helix [28]. Each domain includes thirteen α-Spiral, fifteen β-Folding and eight peptide chains.

### Enzymatic properties of purified AlaDH

The reduction reaction activity of AlaDH was firstly evaluated (Table 1). Among the four different AlaDHs, BcAlaDH exhibited obviously higher reduction activity than the other three AlaDHs. However, the affinity of L-alanine for BsAlaDH was relatively low indicated by the highest *K*m value of 2.25 ± 0.40 mM. The maximum catalytic efficiency of AlaDH reduction reaction is 37.92 ± 6.76 s^−1^ mM^−1^ achieved by BsAlaDH.

**Table 1:** Kinetic parameters of AlaDH in reductive and oxidative reaction

	Specific activity (U/mg)	*K*_m_ (mM)	*V*_max_ (μmol/min)	*k*_cat_ (s^−1^)	*k*_cat_/*K*_m_ (s^−1^ mM^−1^)
Reductive reaction					
BsAlaDH	5.22 ± 0.03	2.25 ± 0.40	64.70 ± 6.34	36.34 ± 0.18	16.68 ± 3.42
BcAlaDH	5.64 ± 0.34	1.10 ± 0.15	66.91 ± 3.70	40.43 ± 2.93	37.92 ± 6.76
MsAlaDH	4.48 ± 0.62	1.69 ± 0.22	23.69 ± 1.52	28.28 ± 4.39	17.02 ± 4.00
GsAlaDH	2.00 ± 0.06	0.81 ± 0.25	10.70 ± 1.07	11.94 ± 0.06	16.29 ± 5.81
Oxidative reaction					
BsAlaDH	92.20 ± 5.94	0.07 ± 0.03	148.30 ± 2.10	103.97 ± 1.77	1819.48 ± 10.25
BcAlaDH	72.54 ± 1.12	1.10 ± 0.08	537.90 ± 1.15	122.33 ± 2.19	111.80 ± 9.67
MsAlaDH	87.50 ± 2.00	0.47 ± 0.03	48.09 ± .518	136.72 ± 1.95	292.09 ± 2.06
GsAlaDH	31.30 ± 0.42	0.23 ± 0.03	193.50 ± 2.37	108.67 ± 2.13	480.66 ± 7.22

The pyruvate reductive aminase activity of four AlaDHs was then investigated, and the results are also shown in Table 1. BsAlaDH and BcAlaDH exhibited higher enzyme activity than the other two AlaDHs. In addition, BsAlaDH possessed strongest binding capacity with substrate pyruvate indicated by the *K*m value of 0.07 ± 0.03 mM. This *K*m value for pyruvate was lower than most reported AlaDHs, such as *M. tuberculosis* (2.8 mM) [29], *Halobacterium salinarum* R1 (0.95 mM) [30], *Thermus caldophilus* (0.75 mM) [31] and *Phormidium lapideum* (0.33 mM) [32]. In addition, BsAlaDH achieved a highest catalytic efficiency indicated by *K*_cat_/*K*_m_ of 1819.48 ± 10.25 s^−1^ mM^−1^. Interestingly, BcAlaDH exhibited similar affinity for L-alanine and pyruvate, which is clearly different for the other three AlaDHs.

In addition, the pure enzyme solutions of BsAlaDH, BcAlaDH, MsAlaDH and GsAlaDH were collected after purification, and their protein concentration were determined by BCA method. As shown in Table 2, the protein concentration of BcAlaDH could reach 5.27 ± 0.33 mg/mL, which is much higher than the other three AlaDHs. Consistent with protein concentration, BcAlaDH also exihibited the highest total enzyme activity of 496.80 ± 1.26 U.

**Table 2:** Total enzyme activity and protein concentration of AlaDH

	Volume (mL)	Toal enzyme activity (U)	Protein concebtration (mg/mL)
BsAlaDH	10	371.15 ± 2.51	1.98 ± 0.21
BcAlaDH	10	496.80 ± 1.26	5.27 ± 0.33
MsAlaDH	8	350.40 ± 2.01	1.74 ± 0.04
GsAlaDH	7	196.87 ± 1.78	1.75 ± 0.06

### Optimal reaction temperature and pH

**Fig. 5:**
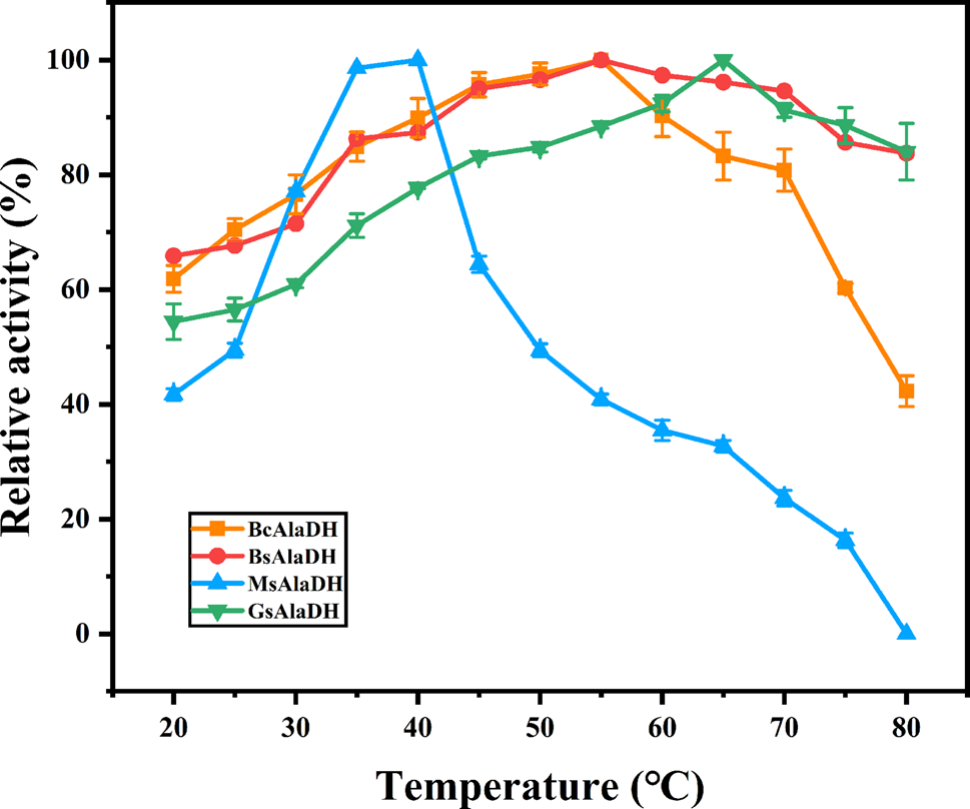
Effect of temperature on enzyme activity

**Fig. 6:**
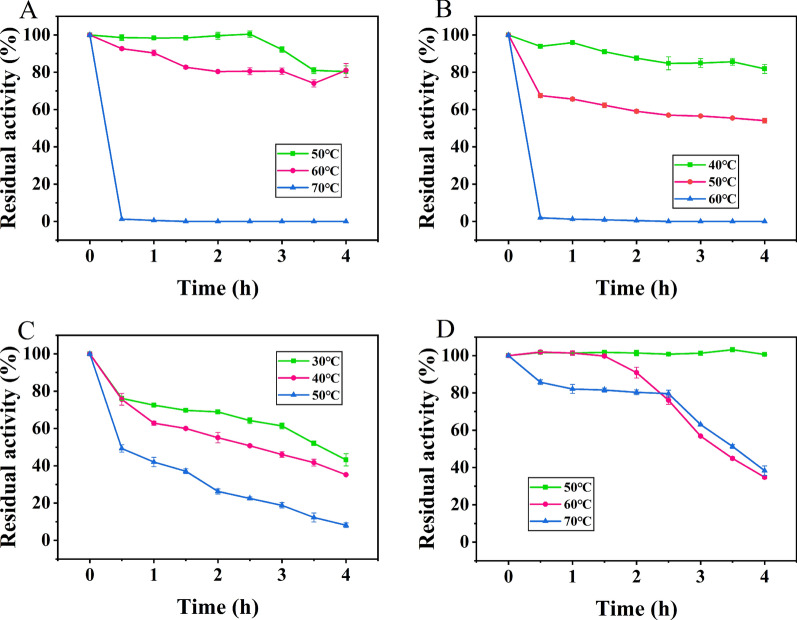
Temperature stability of (**A**) BcAlaDH, (**B**) BsAlaDH, (**C**) MsAlaDH and (**D**) GsAlaDH

The effect of temperature on enzyme activity was then studied by measuring the ammoniation activity of AlaDH at different temperatures (20–80 ℃). As shown in Figs. 5, 6, BsAlaDH exhibited a maximum activity for pyruvate as the substrate at 55 ℃. After incubation at 40–50 ℃ for 4 h, the enzyme activity of AlaDH can be maintained at more than 50%. In contrast, after incubation at 60 ℃ for 0.5 h, the enzyme activity of BsAlaDH was completely lost. Compared with BsAlaDH, the relative enzyme activity of BcAlaDH was maintained nearly 100% during incubation at 50 ℃ before 2.5 h. This enzyme stability at 50 ℃ was also exhibited for GsAlaDH. Especially, the optimum temperature of GsAlaDH was 65 ℃, and it can maintain 100% activity after incubation at 50 ℃ for 4 h. In addition, even incubation at 70 ℃ for 2.5 h, GsAlaDH can still maintain more than 80% activity. Compared with most AlaDHs, such as those from *Rhodobacter capsulatus*, *M. tuberculosis* and *Enterbacter aerogenes* with optimum temperature of 30 ℃, 22 ℃ and 30 ℃, respectively [2, 25, 33], the heat-resistant characteristic of GsAlaDH was excellent, only second to TtAlaDH and AfAlaDH with optimum catalytic temperature of 86 ℃ and 82 ℃ separately [34]. This suggested a potential application value for GsAlaDH in relatively high temperature environment.

**Fig. 7:**
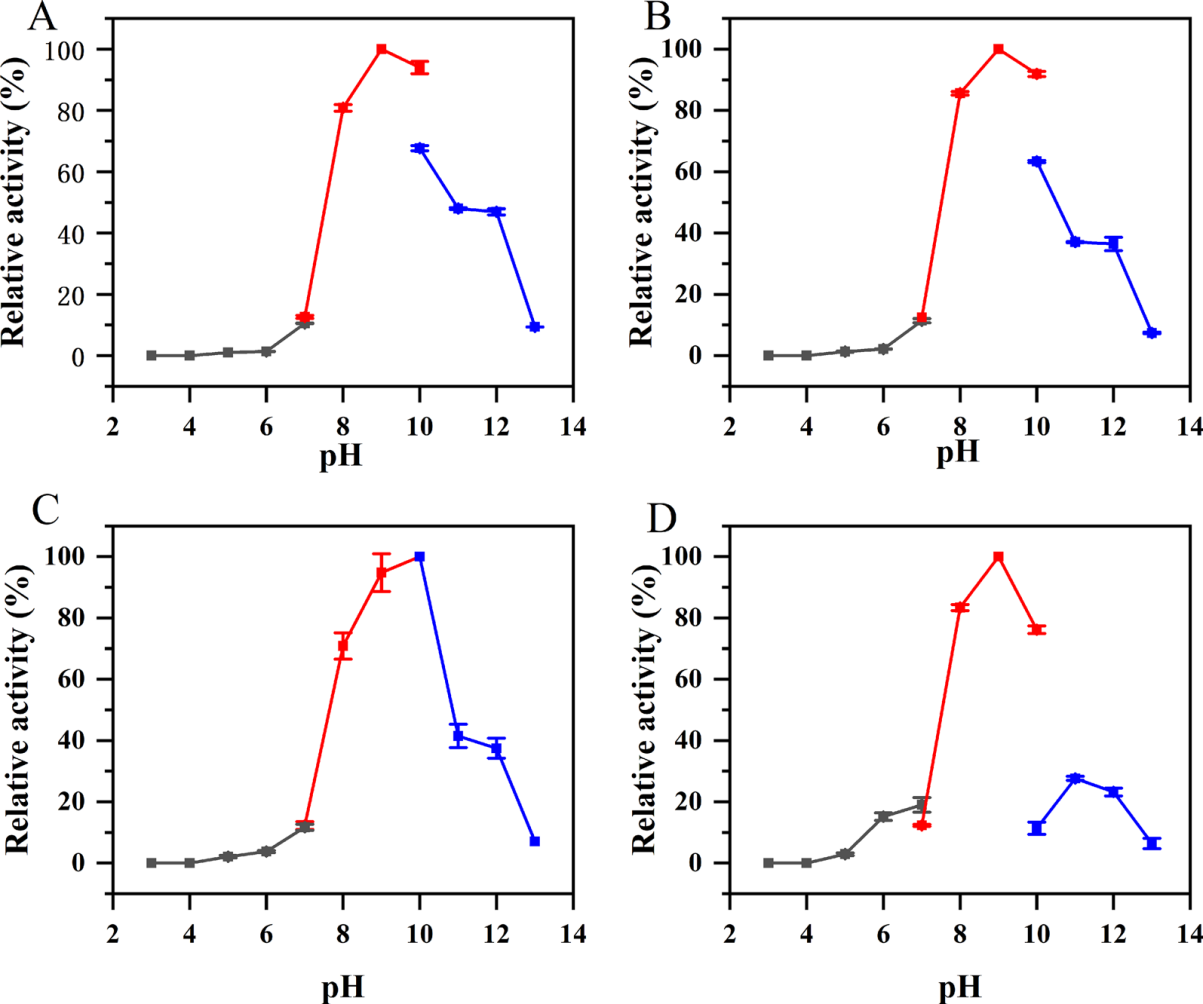
Effect of pH on enzyme activity of (**A**) BcAlaDH, (**B**) BsAlaDH, (**C**) MsAlaDH and (**D**) GsAlaDH. The enzyme activity was detected using pyruvic acid as a substrate. The following buffers were used: 0.1 M citrate (pH 3.0–7.0), 0.1 M Tris–HCl (pH 7.0–10.0) and 0.1 M glycine- NaOH (pH 10.0–13.0)

The effect of pH on enzyme activity was studied by measuring the enzyme activity of AlaDH at different pH (3.0–13.0). As shown in Fig. 7, the optimal pH of BcAlaDH, BsAlaDH and GsAlaDH are all 9.0, which were consistent with BcAlaDH reported by Porumb et al. [35]. In contrast, the optimal catalytic pH of MsAlaDH is 10.0, similar with AlaDH from *Bacillus pseudofirmus* [20].

**Fig. 8:**
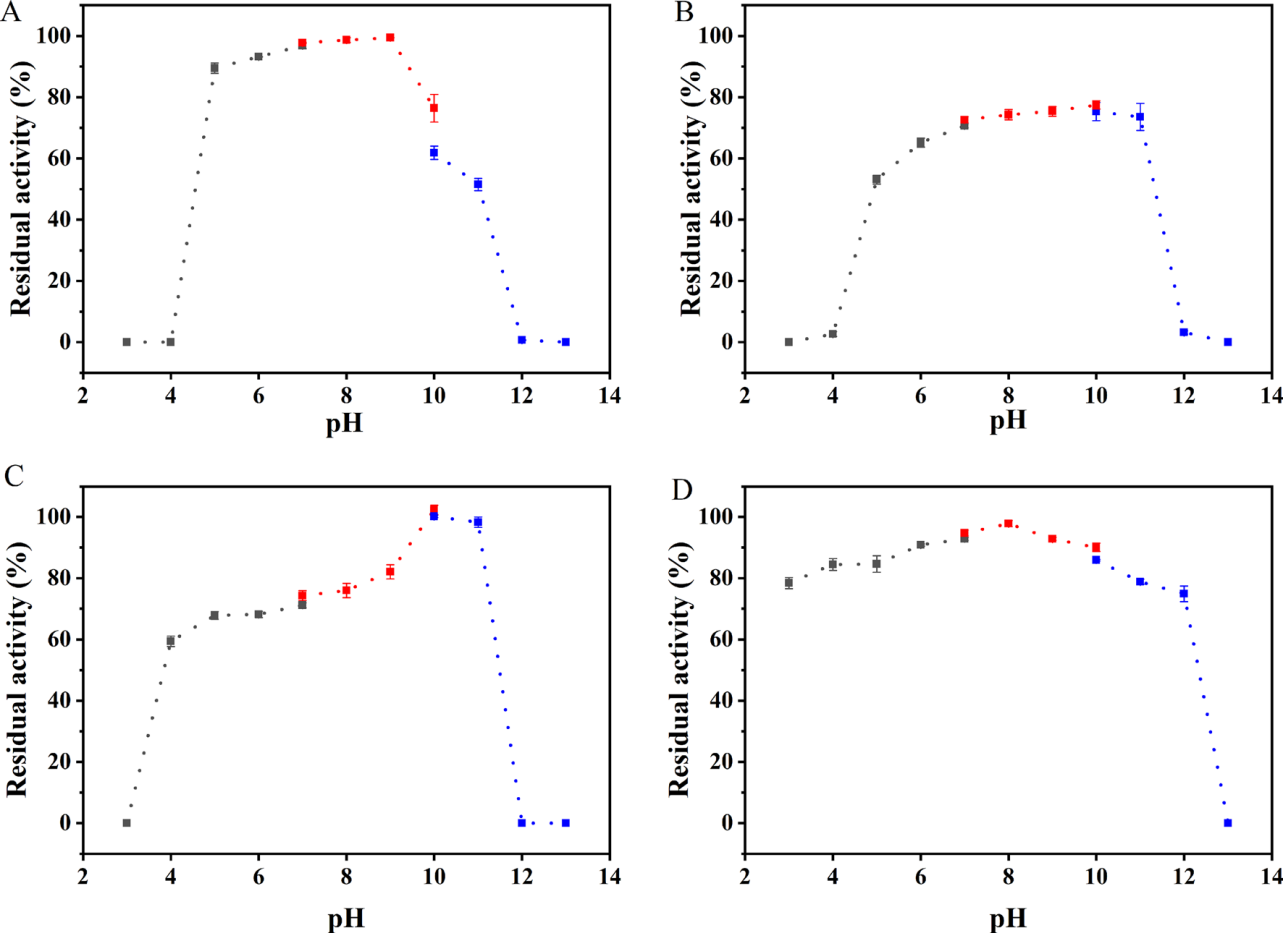
Effect of pH on stability of (**A**) BcAlaDH, (**B**) BsAlaDH, (**C**) MsAlaDH and (**D**) GsAlaDH

Then, the pH stability of four AlaDH was explored. As shown in Fig. 8, for BcAlaDH and BsAlaDH, the enzyme activity was completely lost after incubation in strong acid or alkali environment (pH 3.0–4.0 or pH 12.0–13.0) for 1 h. In contrast, MsAlaDH and GsAlaDH exhibited particular pH tolerance than the other two AlaDHs. Especially for GsAlaDH, more than 70% of the enzyme activity can be retained even incubation at pH 3.0–12.0 for 1 h. Compared with previous reported AlaDH from *Helicobacter aurati* [36], the pH stability of GsAlaDH was even stronger, indicating its potential application value in relatively extreme pH.

### Effects of metal ions and chemicals

The influence of different metal ions and chemicals on four AlaDHs was then investigated. As shown in Table 3, Zn^2+^ and Cu^2+^ exhibited strongest inhibitory effect on AlaDH. When 5 mM of Cu^2+^ was added into the reaction system, only about 1% of AlaDH enzyme activity was retained. Strong inhibition effect of Cu^2+^ and Zn^2+^ were also observed for AlaDH from *Helicobacter aurati* [36]. In contrast, Na^+^ and K^+^ could slightly increase the AlaDH activity less than 14%. For other metal ions apart from Na^+^ and K^+^, the inhibitory effect on AlaDH is significantly strengthened when the concentration of metal ions increases from 1 to 5 mM. Mercaptoethanol and DTT also exhibited a little positive effect on enzyme activities for all of four AlaDHs, which is consistent with the previous research results [2]. This phenomenon may be resulted from a potential protective effect on the mercapto groups of AlaDH.

**Table 3:** Effects of different metal ions on AlaDH

Relative activity (%)								
Metal ion	1 mM				5 mM			
	BsAlaDH	BcAlaDH	MsAlaDH	GsAlaDH	BsAlaDH	BcAlaDH	MsAlaDH	GsAlaDH
Control	100.00 ± 0.90	100.00 ± 0.80	100.00 ± 2.20	100.00 ± 1.60	100.00 ± 0.70	100.00 ± 1.20	100.00 ± 0.70	100.00 ± 1.10
Co^2+^	82.60 ± 0.71	50.95 ± 1.20	72.10 ± 1.27	80.80 ± 2.83	26.10 ± 0.28	45.80 ± 0.85	41.65 ± 0.92	54.80 ± 2.40
Cu^2+^	56.65 ± 0.78	34.30 ± 1.13	44.75 ± 0.49	61.80 ± 1.27	5.10 ± 0.14	2.70 ± 1.13	1.50 ± 0.42	6.10 ± 1.84
Fe^2+^	87.45 ± 0.78	31.35 ± 1.06	55.20 ± 0.57	83.20 ± 1.84	53.70 ± 0.71	14.55 ± 0.47	29.30 ± 1.27	51.65 ± 2.19
Ca^2+^	87.80 ± 1.27	77.80 ± 2.12	87.65 ± 0.64	87.80 ± 1.56	64.30 ± 0.71	53.30 ± 1.70	63.85 ± 0.35	63.90 ± 1.56
Mg^2+^	78.05 ± 1.06	38.05 ± 0.21	96.95 ± 0.78	104.65 ± 3.04	32.70 ± 0.57	56.60 ± 0.99	86.05 ± 0.21	101.5 ± 0.85
Mn^2+^	81.95 ± 0.07	98.05 ± 1.63	92.55 ± 0.78	84.60 ± 0.57	39.25 ± 1.20	60.95 ± 0.35	79.60 ± 1.84	55.65 ± 4.03
Zn^2+^	2.15 ± 0.21	16.05 ± 0.07	8.85 ± 0.35	11.25 ± 1.34	0.00 ± 0.00	1.10 ± 0.28	0.05 ± 0.01	1.05 ± 0.21
Na^+^	103.00 ± 0.28	101.35 ± 0.49	96.25 ± 1.77	113.70 ± 1.98	96.45 ± 1.06	97.60 ± 2.40	94.50 ± 1.27	114.00 ± 2.12
K^+^	103.35 ± 1.63	101.55 ± 0.78	100.25 ± 2.69	105.25 ± 1.34	107.60 ± 1.84	108.55 ± 1.20	103.80 ± 0.42	111.10 ± 2.26
EDTA	63.75 ± 1.63	98.70 ± 1.84	95.05 ± 0.49	83.50 ± 2.26	68.55 ± 0.78	84.40 ± 0.14	78.30 ± 0.99	64.00 ± 1.56
Mercaptoethanol	103.40 ± 1.32	104.25 ± 1.32	102.25 ± 1.55	106.10 ± 1.02	104.75 ± 1.66	105.25 ± 2.05	105.50 ± 1.82	107.10 ± 1.35
DTT	96.60 ± 0.71	98.15 ± 1.20	108.80 ± 0.42	115.35 ± 2.19	108.45 ± 0.44	100.15 ± 1.20	125.25 ± 2.19	110.15 ± 1.20

### Application of AlaDHs in L-alanine synthesis of wild E. coli

**Fig. 9:**
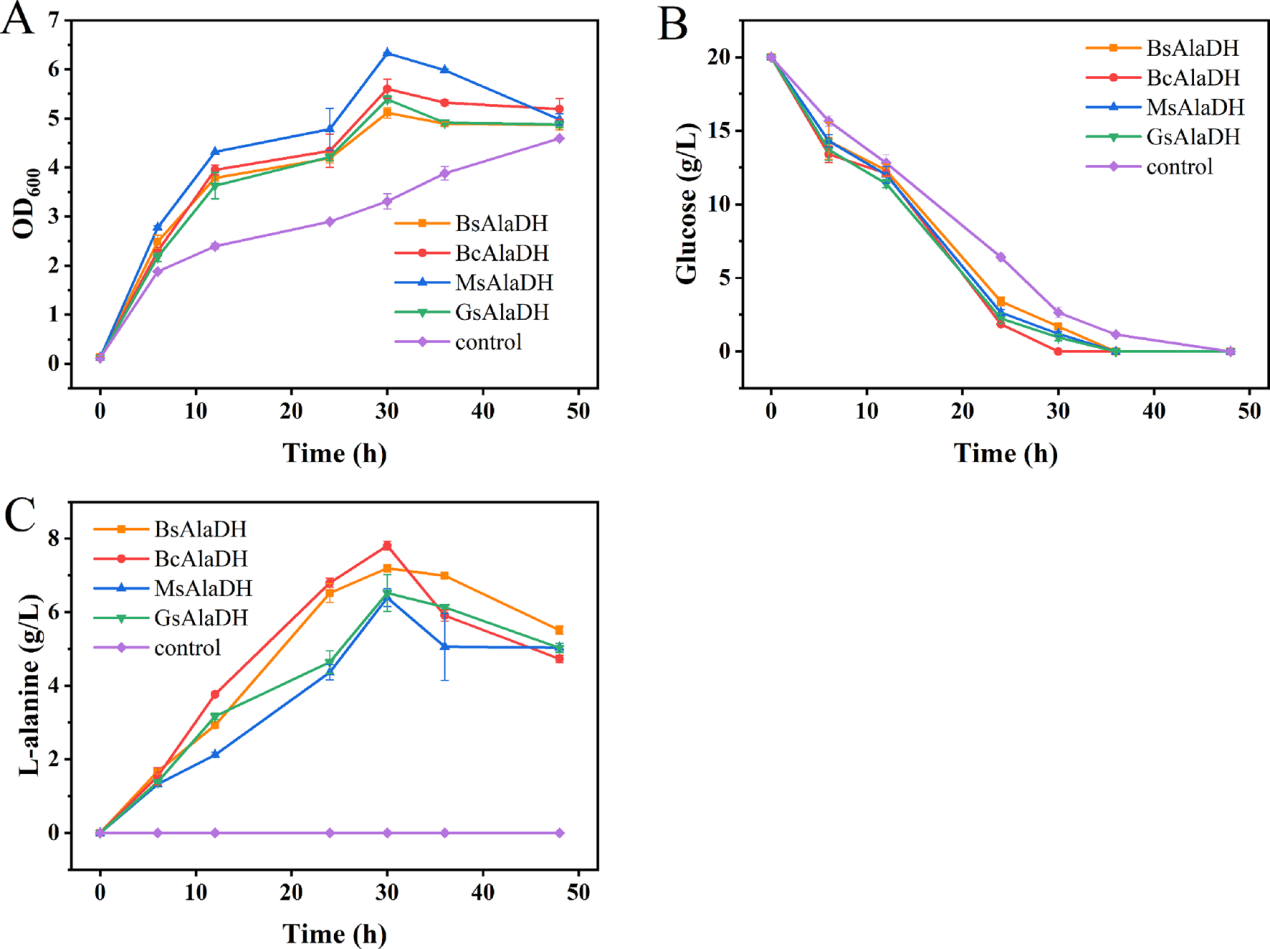
Batch fermentation of *E. coli* BL21(DE3) with or without BsAlaDH, BcAlaDH, MsAlaDH or GsAlaDH respectively. **A** strain growth; **B** glucose consumption; **C** L-alanine titer. The error bars represent standard deviations from three replicate fermentations

The effect of BsAlaDH, BcAlaDH, MsAlaDH and GsAlaDH on L-alanine production in wild *E. coli* was investigated. These four AlaDHs on plasmid pET28a were transformed into *E. coli* BL21(DE3), respectively, and batch fermentation was then carried out. As shown in Fig. 9, the control strain containing empty pET28a exhibited a little worse growth and slower glucose consumption rate than the other four recombinant strains, suggesting no obvious metabolic burden was generated for host *E. coli* after overexpression of AlaDH. In addition, no L-alanine was accumulated for control strain, indicating no endogenous AlaDH exists in *E. coli* BL21(DE3). In contrast, 7.19 g/L, 7.81 g/L, 6.39 g/L and 6.52 g/L of L-alanine were detected in BL21 strains from 20 g/L glucose by employing BsAlaDH, BcAlaDH, MsAlaDH and GsAlaDH, respectively. This further demonstrated the applicatiion potentail of these four AlaDHs in the production of L-alanine production. In addition, the L-alanine titer of all four recombinant strains began to decrease after 30 h, probably due to the deplete of glucose. This phenomenon was also reported by Wada et al. [37].

### Construction of an L-alanine producing strain based on different AlaDHs

**Fig. 10:**
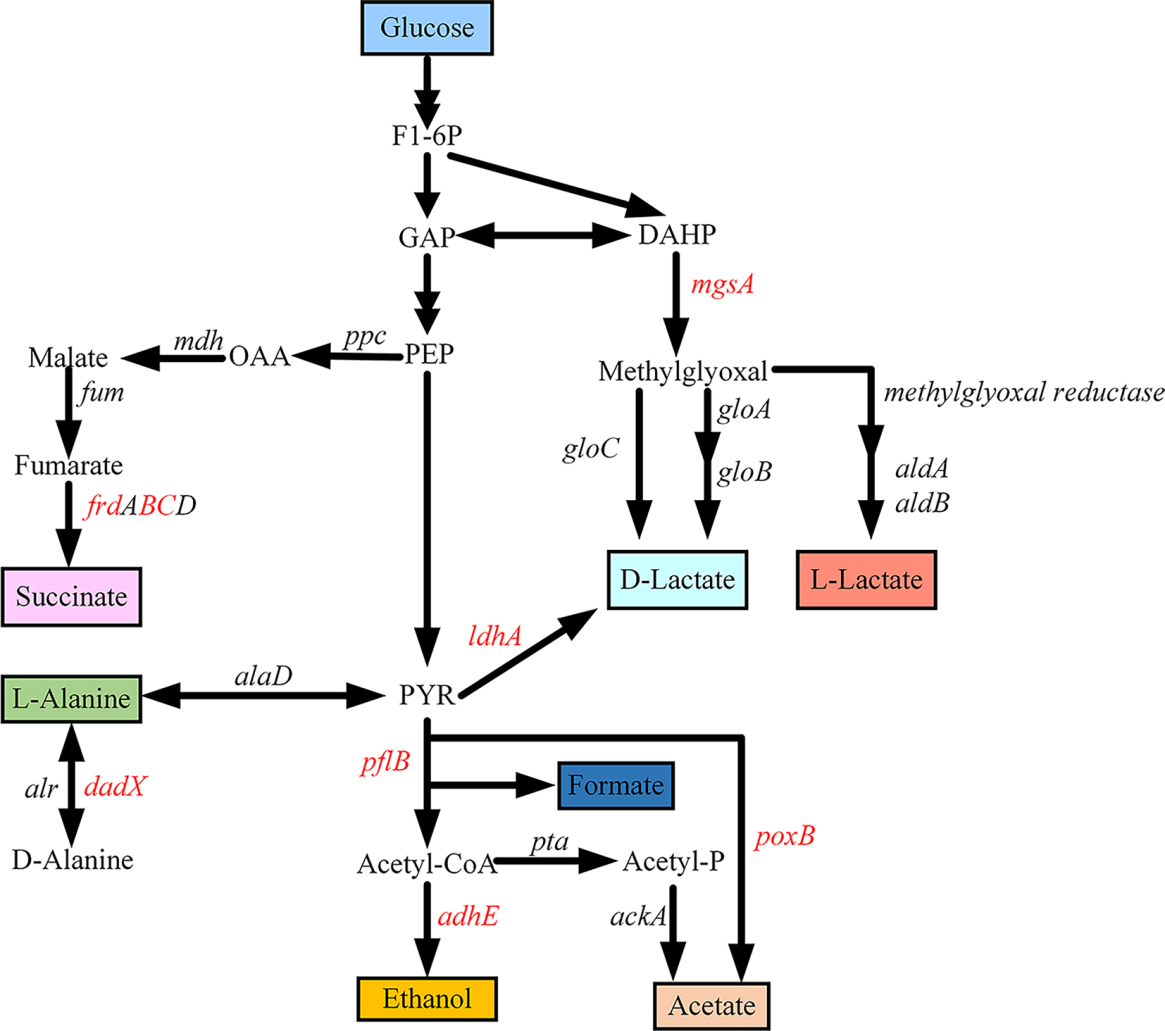
L-alanine pathway in recombinant *E. coli*. Deletions of native genes in *E. coli* was indicated in red color

To further improve the L-alanine production in* E. coli*, carefully engineering of L-alanine synthetic and competitive pathway were necessary. To facilitate strain construction and save engineering time, previously constructed M-1 strain was selected as a base strain. In M-1 strain, *ldhA* encoding D-lactate dehydrogenase, *pflB* encoding pyruvate formate lyase, *poxB* encoding pyruvate oxidase and *adhE* encoding ethanol dehydrogenase was deleted in turn, thereby blocking carbon flows directed into formate, acetate, ethanol and lactate (Fig. 10). Apart from *ldhA*, the methylglyoxal bypass is another pathway that produces lactate in *E. coli* [38]. Deletion of the *mgsA* gene encoding methylglyoxal synthase has been shown to improve biomass accumulation [39]. In addition, *frdBC* gene encoding fumarate reductase was then inactivated to save more PEP for generation of pyruvate.

**Fig. 11:**
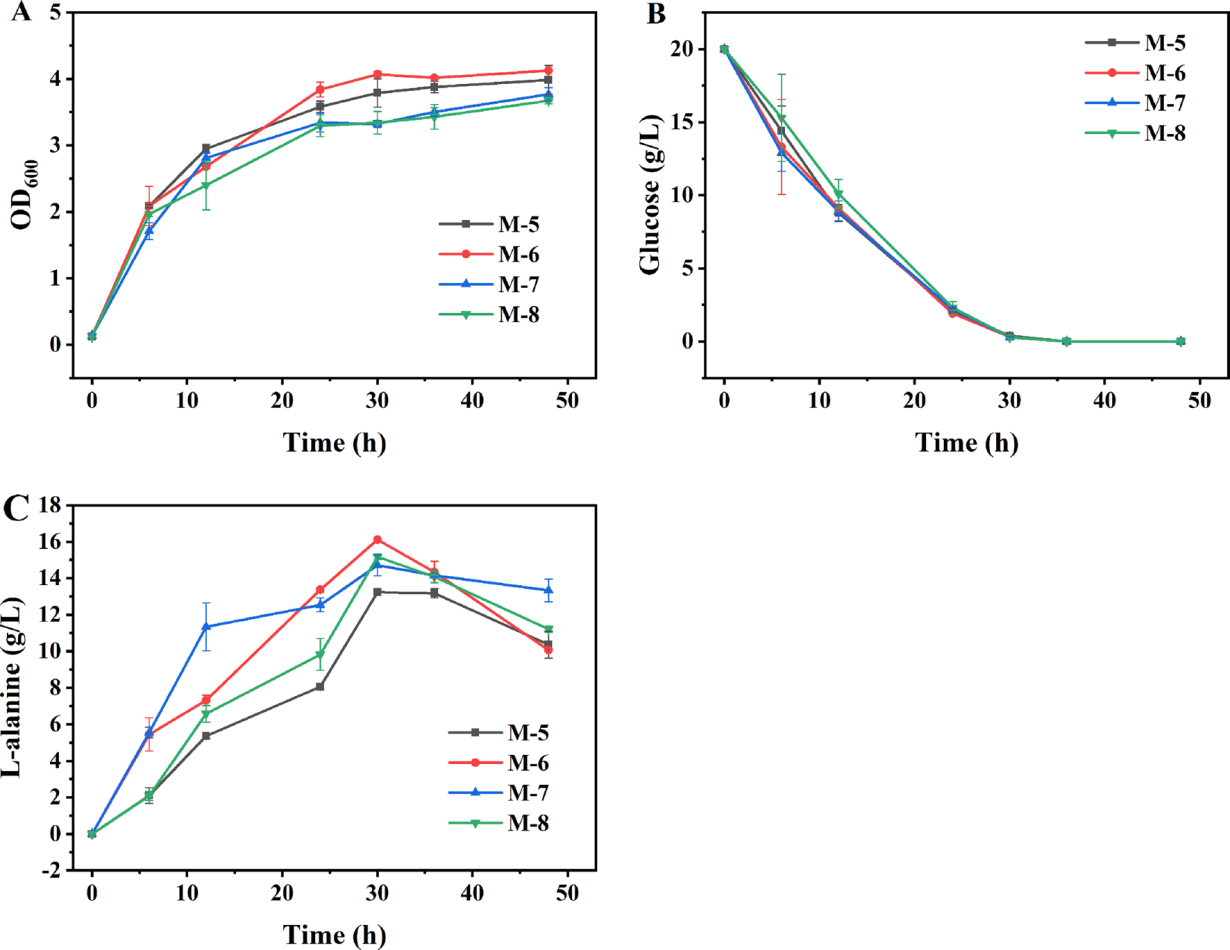
Batch fermentation of recombinant *E. coli* M-5, M-6, M-7 and M-8. **A** strain growth; **B** glucose consumption; **C** L-alanine titer. The error bars represent standard deviations from three replicate fermentations

There are two distinct alanine racemase in *E. coli*, Alr and DadX, which are responsible for the transformation between L-alanine and D-alanine. Among them, DadX contributes most of intracellular racemase activity. Accordingly, deletion of *dadX* gene in *E. coli* is benefit for improving of the chiral purity of L-alanine [40]. After deletion of the three genes *mgsA*, *frdBC* and *dadX* in turn in *E. coli* M-1, M-4 strain was obtained. And then, BsAlaDH, BcAlaDH, MsAlaDH, and GsAlaDH were ligated into an expression vector pTrc99a and transformed into M-4, respectively, to generate recombinant *E. coli* strains M-5, M-6, M-7 and M-8. In the batch fermentation, the recombinant strains M-5, M-6, M-7 and M-8 exhibited similar glucose consumption curve and 20 g/L glucose was completely used after 36 h (Fig. 11). M-5 and M-6 exhibited a litter better growth than M-7 and M-8, probably due to different metabolic burden generated by different AlaDHs. M-5, M-6, M-7 and M-8 were able to synthesize 13.23 g/L, 16.11 g/L, 14.71 g/L and 15.17 g/L L-alanine, respectively, at 30 h. When the residual glucose was below 1 g/L, the L-alanine titer began to decrease, which was consistent with *E. coli* BL21(DE3) strains containing four AlaDHs on plasmid pET28a (Fig. 9).

**Fig. 12:**
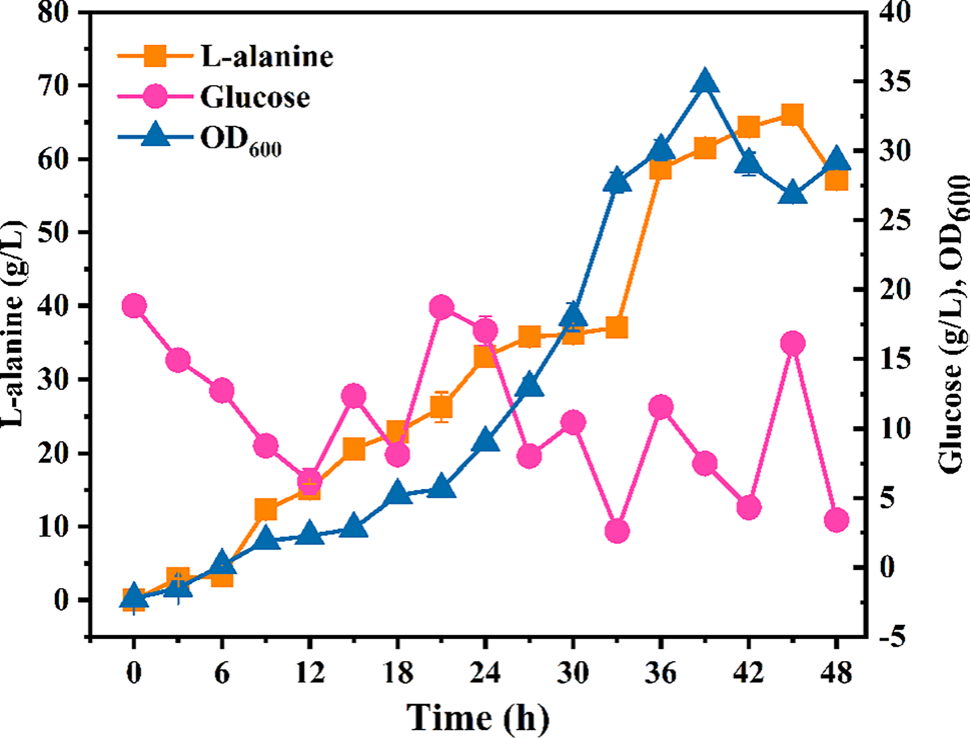
Fed-batch fermentation of *E. coli* M-6. The error bars represent standard deviations from three replicate measurements

To explore the production potential of M-6, which exhibited highest L-alanine titer in batch fermentation, a 5 L fed-batch fermentation was then performed. As shown in Fig. 12, strain M-6 could produce 66 g/L of L-alanine at 45 h, while the glucose concentration was controlled between 5 and 20 g/L. The NADH generated in glycolysis is necessary for the reduction of pyruvate to L-alanine via L-alanine dehydrogenase. As NADH can be consumed by both oxidative phosphorylation and the enzyme NADH oxidase in normal aerobic fermentation, controlling oxygen supply may be benefit for L-alanine production.

**Fig. 13:**
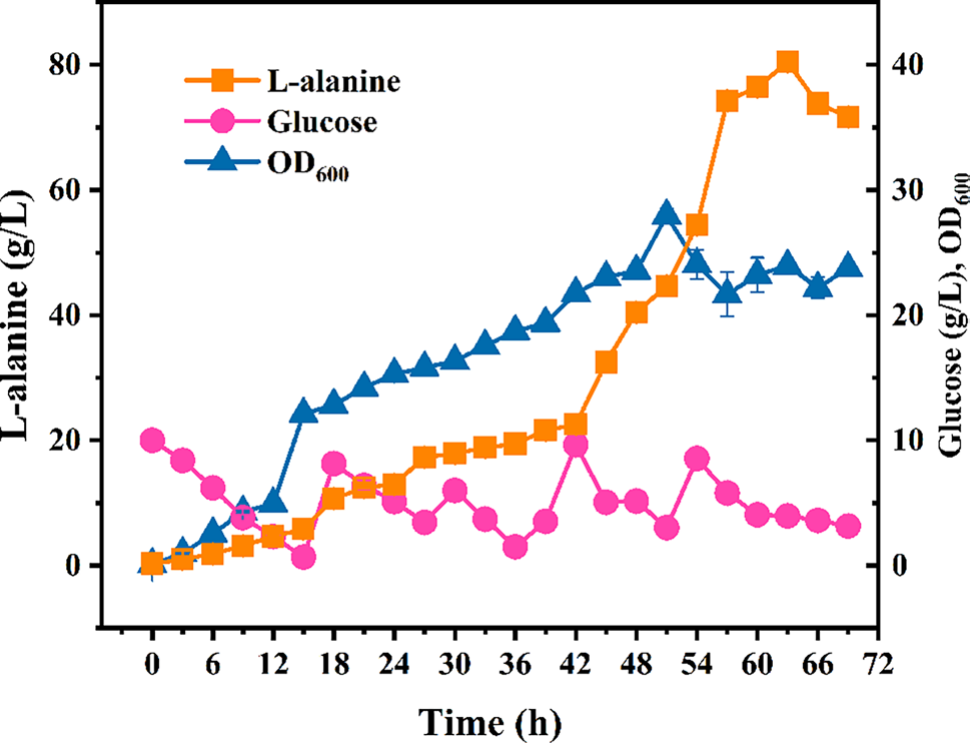
Fed-batch fermentation of *E. coli* M-6 under oxygen limited conditions. The error bars represent standard deviations from three replicate measurements

Thus, an oxygen limited fed-batch fermentation was carried out for M-6. The pH and dissolved oxygen (DO) profiles was exhibited in Additional file 1: Fig. S2, S3. When the cell concentration reached an OD_600_ of approximately 25–30, the air supplement was stopped and the agitation was reduced to 200 rpm. As shown in Fig. 13, the maximum L-alanine titer could further increase to 80.46 g/L with a yield of 1.02 g/g glucose, representing the highest yield for microbial L-alanine production using glucose as sole carbon source.

To facilitate a comparison between M-6 with other strains, previously constructed L-alanine-producing *E. coli* strains with relative high titers were exhibited in Additional file 1: Table S1. Zhang et al. constructed a recombinant *E. coli* XZ132 by blocking formate, acetate, lactate, ethanol and D-alanine synthetic pathways and integrated AlaDH from *G.stearothermophilus* into the genome. As a result, XZ132 could produce 114 g/L L-alanine [40]. In addition, a thermo-regulated genetic switch was applied to dynamically control the expression of AlaDH from *G. stearothermophilus* in *E. coli* B0016-060BC. After 40 h fed-batch fermentation, the resulting strain could generate a maxmium L-alanine titer of 120.8 g/L [41]. Accordingly, to further increasing the L-alanine production titer of M-6, strain engineering such as inactivation of AckA-Pta acetate synthetic pathway and optimization of fed-batch fermentation conditions such as glucose supplement strategies can be carried out. In addition, screening of novel robust AlaDHs or engineering of existing AlaDHs with high enzyme activities are also benefit for L-alanine production in *E. coli*.

## Conclusions

In this study, enzyme assay, biochemical characterization and structure analysis of BsAlaDH, BcAlaDH, MsAlaDH and GsAlaDH were firstly carefully explored. In addition, GsAlaDH showed an excellent temperature and pH tolerance than the other three AlaDHs, demonstrating a robust characteristic for application in complex and hostile environment. Lastly, AlaDHs explored in this work were employed for L-alanine production from glucose in *E. coli*. All of four AlaDHs could achieve a success accumulation of L-alanine and M-6 with BcAlaDH could achieve 80.46 g/L L-alanine accumulation in 5 L fed-batch with a yield of 1.02 g/g glucose, representing the highest yield for microbial L-alanine production using glucose as sole carbon source.

## Materials and methods

### Strains, plasmid and chemicals

All strains, plasmids, and oligonucleotides used in this study are listed in Tables 4–5. *E. coli* BL21(DE3) and DH5α were employed for protein expression and DNA manipulation separately. Previous constructed *E. coli* M-1 strain (unpublished data) was selected as a base strain for constructing L-alanine producing strain. The expression plasmid pET28a (Novagen) was used for protein expression. The plasmid pTrc99a was employed for AlaDH overexpression in L-alanine producing *E. coli* strain. T4 DNA ligases were purchased from NEB (Beijing, China) and restriction endonucleases were obtained from ThermoFisher (Shanghai, China). DNA extraction kit, plasmid extraction kit and His-tag protein purification kit were all purchased from Omega (San Diego, CA, USA). Antibiotics, isopropyl-β-dithiogalactoside (IPTG) and other commonly used molecular biology reagents were purchased from Solarbio (Beijing, China) and Sangon Biotech (Shanghai, China).

**Table 4:** Strains and plasmids used in this study

Strains	Relevant genotype	Reference
DH5α	*F*^*−*^*, φ80dlacZ, ΔM15, Δ(lacZYA-argF), U169, recA1, endA1, hsdR17(rK– mK*^+^*), phoA, supE44, λ*^*−*^*, thi–1, gyrA96, relA1*	Lab stock
BL21(DE3)	*F-*, *omp*T, *hsdS*_*B*_* (r*_*B*_*-, m*_*B*_*-)*, *gal*, *dcm*, (DE3)	Lab stock
M-1	BW25113(Δ*pflB*Δ*poxB*Δ*adhE*Δ*ldhA*)	Unpublished data
M-2	M-1(Δ*mgsA*)	This study
M-3	M-2(Δ*frdBC*)	This study
M-4	M-3(Δ*dadX*)	This study
M-5	M-4(pQ-1)	This study
M-6	M-4(pQ-2)	This study
M-7	M-4(pQ-3)	This study
M-8	M-4(pQ-4)	This study
Plasmids		
pET28a	*kan*, expression plasmid	Lab stock
pN-1	pET28a-Bs*ald*	This study
pN-2	pET28a-Bc*ald*	This study
pN-3	pET28a-Ms*ald*	This study
pN-4	pET28a-Gs*ald*	This study
pKD3	*bla*, FRT-*cat*-FRT	[43]
pKD4	*bla*, FRT-*kan*-FRT	[43]
pCP20	*bla* and *cat*, helper plasmid	[45]
pTKRed	*spc*, helper plasmid	[44]
pTrc99a	*bla*, cloning vector	Lab stock
pQ-1	pTrc99a-Bs*ald*	This study
pQ-2	pTrc99a-Bc*ald*	This study
pQ-3	pTrc99a-Ms*ald*	This study
pQ-4	pTrc99a-Gs*ald*	This study

**Table 5:** Primers used in this study

Name	Sequence (5’-3’)
Bcald-F	CGCGGATCCATGCGTATTGGGGTACCAGC
Bcald-R	CGAGCTCTTAGCAAGATACTGTTTC
Bsald-F	CGCGGATCCATGATCATAGGGGTTCCTAA
Bsald-R	CGAGCTCTTAAGCACCCGCCACAGATG
Gsald-F	CCGGAATTCATGATTATTGGAGTGCCAAAG
Gsald-R	CCCAAGCTTTTAGTTGGCGGCCAACGTTTT
Msald-F	CCGGAATTCATGCTCGTCGGAATCCCGACCGAGAT
Msald-R	CCCAAGCTTTTACGCCAGGAACTGTGCCGC
mgsA-QF	CGATAAGTGCTTACAGTAATCTGTAGGAAAGTTAACTACGGATGTACATTGTGTAGGCTGGAGCTGCTTC
mgsA-QR	GGTGGCGAGAAAACCGTAAGAAACAGGTGGCGTTTGCCACCTGTGCAATAATGGGAATTAGCCATGGTCC
mgsA-JF	ACGCTGCTTTCGGGTGTTTCCGAGCTGGAT
mgsA-JR	TTACGTCATCATCGTTGGCTTGCAGGAGGGAAGTGA
frdBC-QF	ATGCAGCCGATAAGGCGGAAGCAGCCAATAAGAAGGAGAAGGCGAGTGTAGGCTGGAGCTGCTTCGAAGT
frdBC-QR	GGTTCGTCAGAACGCTTTGGATTTGGATTAATCATCTCAGGCTCCATGGGAATTAGCCATGGTCCATATG
frdBC-JF	TATGGCGCACTCCGCAATGGCACGTAAAGA
frdBC-JR	ATACGGTGTAAACCACACCACAGCGGCAGA
dadX-QF	CGCCATCACGTCCGGGCCATTTACATGGCGCACACAGCTAAGGAAACGAGGTGTAGGCTGGAGCTGCTTC
dadX-QR	TGATTTTTTTGCACCCAGAAGACGTTGCCTCCGATCCGGCTTACAACAAGATGGGAATTAGCCATGGTCCATATG
dadX-JF	CCGGTTGTCGGGCGTACACGCTTTAAAAAT
dadX-JR	CACAAATCTATGTACAGGCTCCATCAAGGG
Bsald(99a)-F	CGAGCTCATGATCATAGGGGTTCCTAAAGAGA
Bsald(99a)-R	CGCGGATCCTTAAGCACCCGCCACAGATGATTCA
Bcald(99a)-F	CGAGCTCATGCGTATTGGGGTACCAGCAGAAA
Bcald(99a)-R	CGCGGATCCTTAGCAAGATACTGTTTCTGCTTCTAATAA
Msald(99a)-F	CCGGAATTCATGCTCGTCGGAATCCCGACCGAGATCAAG
Msald(99a)-R	CGCGGATCCTTACGCCAGGAACTGTGCCGCGTCGGTGAA
Gsald(99a)-F	CCGGAATTCATGAAGATCGGCATTCCAAAAGAAATCAAA
Gsald(99a)-R	CGCGGATCCTCATCCCTGCAGCAACGAAT

### Expression and protein purification of AlaDH

The *ald* gene encoding AlaDH derived from *B. subtilis* 168, *B. cereus*, *M. smegmatis* MC^2^ 155 and *G. stearothermophilus* were directly synthesized by Tsingke Biotechnology (Beijing, China), respectively. And then, these synthesized genes ligated into pUC19 were used as PCR templates, and Bsald-F/Bsald-R, Bcald-F/Bcald-R, Msald-F/Msald-R and Gsald-F/Gsald-R were employed as primer pairs, respectively. Afterwards, the expression plasmid pET28a and the target fragments Bs*ald*, Bc*ald*, Ms*ald* and Gs*ald* were double-digested with restriction enzymes *Sac*I/*Bam*HI, *Sac*I/*Bam*HI, *Eco*RI/*Hin*dIII and *Eco*RI/*Hin*dIII, respectively. And then, four gene fragments and linearized plasmid were ligated with T4 DNA ligase at 25 ℃ for 1 h, and the ligation products were transformed into *E. coli* DH5α, respectively. Positive colons containing expected recombinant plasmids were verified by DNA sequencing in Tsingke Biotechnology (Beijing, China).

The successfully constructed recombinant plasmids pET28a-Bs*ald*, pET28a-Bc*ald*, pET28a-Ms*ald* and pET28a-Gs*ald* were transformed into BL21(DE3) competent cells, and then the recombinant strains were cultured with kanamycin (50 μg/mL) in LB liquid medium at 37 ℃ and 200 rpm. Afterwards, 3 mL of seed solution was inoculated into 300 mL fresh LB medium at 37 ℃ and 200 rpm, and 50 mg/mL IPTG were added into the medium when OD_600_ reached 0.6. After supplement of IPTG, the culture condition was changed into 20 ℃ and 180 rpm. When *E. coli* cells were cultivated for 12–14 h, the induced cells were collected by centrifugation at 4 ℃ and 6000 rpm for 10 min. Then, the sediment was washed with 25 mM PBS buffer, and ultrasonic breaking instrument was employed to break cell wall and release intracellular AlaDH. The crude enzyme solution was collected after centrifugation at 4 ℃ and 6000 rpm for 10 min.

Then, 10 mM imidazole and 100 mM NaCl were added to the crude enzyme solution, and the whole sample was supplemented into the Ni^2+^ affinity chromatography column. The purified AlaDH proteins were eluted and collected with different concentrations of imidazole (50 mM, 100 mM, 150 mM, 200 mM) mixed with 100 mM NaCl, and then dialyzed with 25 mM PBS buffer to remove imidazole and NaCl. The molecular weight of denatured protein was determined by 12% SDS–polyacrylamide gel electrophoresis (SDS-PAGE) and protein markers (TaKaRa) was used as the standards.

### Enzyme assay and biochemical characterization

The protein concentration was determined using BCA reagent [42]. For determining the pyruvate reductive aminase activity of AlaDH, the reaction buffer contained 1 M Tris–HCl buffer with pH 8.0, 0.1 M pyruvic acid and 2 M NH_4_Cl was used. The NADH cofactor (0.01 M) was added into the reaction buffer and the absorbance value at 340 nm was monitored within 10 min. The enzymatic reaction was initiated by adding purified recombinant AlaDH. For determining the alanine oxidative dehydrogenase activity of AlaDH, 6 mM NAD^+^ and 0.1 M L-alanine was added into the reaction buffer (500 mM Na_2_CO_3_-NaHCO_3_ with pH 10.0). The enzymatic reaction was initiated by adding the purified recombinant AlaDH. The reaction was monitored by spectrophotometric analysis of NADH change at 340 nm within 10 min. An AlaDH unit (U) is defined as the amount of enzyme required to produce or consume 1 mmol NADH within 1 min.

To explore the optimal temperature for AlaDH, the reaction mixture (0.1 M pyruvic acid, 0.01 M NADH and 2 M NH_4_Cl) containing different AlaDH was incubated at different temperatures (20–80 ℃) and pH 8.0 for 10 min. The enzyme activity was followed by measuring absorbance value at 340 nm by a spectrophotometer (Metash, Shanghai, China). The optimal pH of AlaDH was determined at 37 ℃ using 0.1 M citrate (pH 3.0–7.0), 0.1 M Tris–HCl (pH 7.0–10.0) or 0.1 M glycine-NaOH (pH 10.0–13.0) buffer, respectively. For determining the temperature stability of AlaDH, four AlaDH proteins were incubated at different temperatures for 4 h, and the residual enzyme activity was measured every 0.5 h. For the stability of pH, the purified AlaDH was incubated in 0.1 M citrate (pH 3.0–7.0), 0.1 M Tris–HCl (pH 7.0–10.0) or 0.1 M glycine- NaOH (pH 10.0–13.0) buffer at 4 ℃ for 1 h, and the residual enzyme activity was measured under standard assay. The effects of various chemicals comprising metal ions (Co^2+^, Cu^2+^, Fe^2+^, Ca^2+^, Mg^2+^, Mn^2+^, Ni^2+^, Zn^2+^, Na^+^, K^+^), EDTA, mercaptoethanol and DTT on AlaDH activity were performed at a final concentration of 1 or 5 mM.

### Construction of L-alanine producing strain

In a previous experiment, a recombinant *E. coli* M1 was obtained with deletion of *ldhA* encoding D-lactate dehydrogenase, *pflB* enconding pyruvate formate lyase, *poxB* encoding pyruvate oxidase and *adhE* encoding ethanol dehydrogenase. Based on this strain, *mgsA* gene encoding methylglyoxal synthase, *frdBC* gene encoding fumarate reductase and *dadX* encoding alanine racemase was deleted in turn using one-step gene inactivation method [43]. In brief, plasmid pTKRed containing IPTG induced Red homologous recombinase was firstly transformed into host strain [44]. And then, primers mgsA-QF/mgsA-QR, frdBC-QF/frdBC-QR and dadX-QF/dadX-QR, and template plasmids pKD3 for *mgsA* and pKD4 for *frdBC* and *dadX* were used separately to obtain the linearized DNA flanked by FLP recognition target sites and homologous sequences for target genes. Electroporation was carried out using 50–100 μL electroporation competent cells and 10–100 ng of PCR product. The positive clones on the plates were verified by PCR using the primers mgsA-JF/mgsA-JR, frdBC-JF/frdBC-JR and dadX-JF/dadX-JR, respectively. The chlorampenicol or kanamycin cassette was removed by the helper plasmid pCP20 to obtain *E. coli* strain M-4.

The expression plasmid pTrc99a was employed to overexpress BsAlaDH, BcAlaDH, MsAlaDH and GsAlaDH in M-4. DNA fragments of four AlaDHs was obtained using Bsald(99a)-F/Bsald(99a)-R, Bcald(99a)-F/Bcald(99a)-R, Msald(99a)-F/Msald(99a)-R and Gsald(99a)-F/Gsald(99a)-R as primers, and plasmids pN-1, pN-2, pN-3 and pN-4 as templates. Afterwards, plasmid pTrc99a and the target fragments Bs*ald*, Bc*ald*, Ms*ald* and Gs*ald* were double-digested with restriction enzymes *Sac*I/*Bam*HI, *Sac*I/*Bam*HI, *Eco*RI/*Bam*HI and *Eco*RI/*Bam*HI, respectively. And then, four gene fragments and linearized plasmid were ligated with T4 DNA ligase at 25 ℃ for 1 h, and the ligation products were transformed into *E. coli* DH5α respectively. Positive colons containing expected recombinant plasmids were verified by DNA sequencing in Tsingke Biotechnology (Beijing, China). The resulting plasmids pQ-1, pQ-2, pQ-3 and pQ-4 was transformed into M-4 strain to generate L-alanine producing *E. coli* strain M-5, M-6, M-7 and M-8, respectively.

### Batch and fed-batch fermentation of recombinant E. coli for L-alanine production

For batch fermentation, a single clone was precultured in 50 mL LB medium at 37 °C on a rotary shaker at 200 rpm overnight. One milliliter of overnight culture was inoculated into 50 mL LB medium and cultured for 8–12 h, with 5% (*v*/*v*) seed cultures subsequently inoculated into 100 mL M9-1 fermentation medium containing 2 g/L yeast extract and 20 g/L glucose at 37 °C and 200 rpm [41]. When OD_600_ of recombinant *E. coli* reached 0.6, 50 mg/mL of IPTG was added into the medium and the culture temperature was changed into 30 ℃. Three parallel experiments were conducted for each group. For fed-batch fermentation, a stirred 5 L glass vessel (BIOTECH, Shanghai, China) was used. The inoculum ratio was 3% (*v*/*v*). When glucose concentration in the medium was below 5 g/L, feeding solution containing 500 g/L glucose was added into the medium. The culture temperature was 37 °C, and the pH was controlled at 7.0 with ammonia solution containing 25–28% NH_3_. For an oxygen limited fed-batch fermentation, air was supplied continuously at 1.0–5.0 L/min with an agitation of 200–700 rpm, increasing gradually according to the strain growth to maintain the dissolved oxygen above 30%. When the cell concentration reached an OD_600_ of approximately 25–30, the air supplement was stopped and the agitation was reduced to 200 rpm.

### Analytical methods

Cell growth was monitored as OD_600_ using a UV5100H spectrophotometer (METASH, Shanghai China). Glucose was analyzed by an SBA-40E biosensor (Biology Institute of Shandong Academy of Sciences, China). L-alanine was quantitatively analyzed on a high-performance liquid chromatography equipped with a column of Zorbax Eclipse Plus C18 (250 × 4.6 mm; Agilent, USA). The mobile phase was 0.05 mol/L Na_2_HPO_4_ and methanol (*V*/*V* = 9:1) with a flow rate of 0.8 mL/min. The column was maintained at 30 ℃ and a UV detector was employed at 215 nm with an injection volume of 10 μL.

### Supplementary Information

**Additional file 1: Table S1** Comparison of L-alanine-producing *E. coli* strains with relative high titers. **Fig. S1** Laplace conformation of (A) BsAlaDH, (B) BcAlaDH, (C) MsAlaDH and (D) GsAlaDH. **Fig. S2** The pH profile of *E. coli* M-6 in oxygen limited batch fermentation. **Fig. S3** The dissolved oxygen (DO) profile of *E. coli* M-6 in oxygen limited batch fermentation.

## Data Availability

All data generated and analyzed during this study were included in this manuscript.
