# Supplementary material for: Alanine dehydrogenases from four different microorganisms: characterization and their application in L-alanine production
Source: Biotechnol Biofuels Bioprod. 2023 Aug 3;16:123. doi: 10.1186/s13068-023-02373-5 (PMC10401832; doi:10.1186/s13068-023-02373-5)
Supplement: Supplementary file 1 — Additional file 1: Table S1 Comparison of L-alanine-producing E. coli strains with relative high titers. Fig. S1 Laplace conformation of (A) BsAlaDH, (B) BcAlaDH, (C) MsAlaDH and (D) GsAlaDH. Fig. S2 The pH profile of E. coli M-6 in oxygen limited batch fermentation. Fig. S3 The dissolved oxygen (DO) profile of E. coli M-6 in oxygen limited batch fermentation. [file 13068_2023_2373_MOESM1_ESM.docx]

**Supporting Information**

Alanine dehydrogenases from four different microorganisms: characterization and their application in L-alanine production

Pengfei Gu^&,^ *, Qianqian Ma^&^, Shuo Zhao^&^, Qiang Li, Juan Gao*

School of Biological Science and Technology, University of Jinan, Jinan 250022, People’s Republic of China

*Corresponding author: Pengfei Gu*

Email: bio_gupf@ujn.edu.cn

Tel.: +86-531-82767364

Fax: +86-531-89736818

*Corresponding author: Juan Gao*

Email: bio_gaoj@ujn.edu.cn

Tel.: +86-531-82767364

Fax: +86-531-89736818

^&^These authors contributed equally to this work

Table S1 Comparison of L-alanine-producing *E. coli* strains with relative high titers

| *E. coli* strains | Modified property | Fermentation conditions | Time  (h) | L-alanine titer (g/L) | Yield  (g/g) | Reference |
| --- | --- | --- | --- | --- | --- | --- |
| AL887 (pTrc99A-*alaD*) | *E. coli* (Δ*ldhA*Δ*aceF*) with overexpressed *alaD* from *B. sphaericus* | Two-stage fed-batch process, mineral medium containing glucose, acetic acid, succinic acid, tryptone and yeast extract | 27 | 32 | 0.63 | [1] |
| ALS929(pTrc99A-alaD) | *E. coli* K-12 (Δ*pfl*Δ*pps*Δ*aceEF*Δ*poxB*Δ*ldhA*) pTrc99a-alaD  with overexpressed *alaD* from *B. sphaericus* | Two-phase fed-batch fermentaion containing aerobic cell  growth and anaerobic alanine  production process, mineral medium containing glucose, isoleucine, casamino acid and yeast extract | 48 | 88 | 1 | [2] |
| XZ132 | *E. coli* W (Δ*pfl*Δ*ackA*, Δ*adhE*Δ*mgsA*Δ*dadX*Δ*ldhA*::*alaD* from *Geobacillus stearothermophilus*) | Fed-batch fermentation process, NBS mineral medium containing glucose | 48 | 114 | 0.95 | [3] |
| B0016-060BC | *E. coli* B0016 (Δ*ldhA*Δ*ackA-pta*Δ*pflB*Δ*adhE*Δ*frdA*Δ*dadX*::*cI*ts857-*p*R-*p*L-*alaD*-FRT | Thermoregulated 33 °C aerobic cell growth and 42 °C oxygen-limited alanine production, M9-1 medium containing glucose | 40 | 120.8 | 0.88 | [4] |
| M-6 | BW25113(Δ*pflB*Δ*poxB*Δ*adhE*Δ*ldhA*Δ*mgsA*Δ*frdBC*Δ*dadX*) with overexpressed *alaD* from *B. cereus* | Two-stage fed-batch process, M9-1 medium containing glucose and yeast extract | 63 | 80.46 | 1.02 | This study |


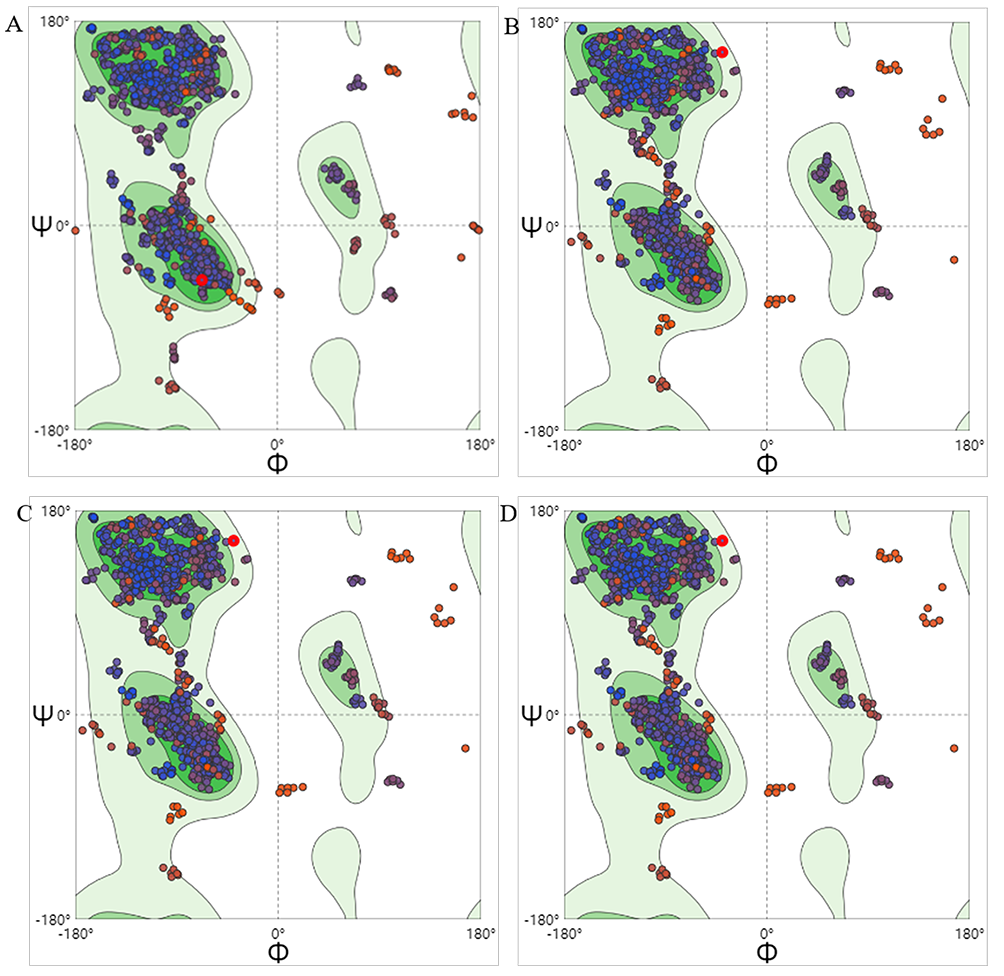


**Fig. S1** Laplace conformation of (A) BsAlaDH, (B) BcAlaDH, (C) MsAlaDH and (D) GsAlaDH





**Fig. S2** The pH profile of *E. coli* M-6 in oxygen limited batch fermentation





**Fig. S3** The dissolved oxygen (DO) profile of *E. coli* M-6 in oxygen limited batch fermentation

**Reference**

1. Lee M, Smith GM, Eiteman MA, Altman E. Aerobic production of alanine by *Escherichia coli aceF ldhA* mutants expressing the *Bacillus sphaericus alaD* gene. Appl Microbiol Biotechnol. 2004;65:56-60.

2. Smith GM, Lee SA, Reilly KC, Eiteman MA, Altman E. Fed-batch two-phase production of alanine by a metabolically engineered *Escherichia coli*. Biotechnol Lett. 2006;28:1695-1700.

3. Zhang X, Jantama K, Moore JC, Shanmugam KT, Ingram LO. Production of L -alanine by metabolically engineered *Escherichia coli*. Appl Microbiol Biotechnol. 2007;77:355-366.

4. Zhou L, Deng C, Cui WJ, Liu ZM, Zhou ZM. Efficient L-alanine production by a thermo-regulated switch in *Escherichia coli*. Appl Biochem Biotechnol. 2016;178:324-337.
